# Comparative Effects of Acute HIIE and MICE on Executive Function, Prefrontal Oxyhaemoglobin Responses, and Related Biomarkers in Sedentary Young Adults: A Randomized Crossover Trial

**DOI:** 10.3390/brainsci16101054

**Published:** 2026-09-30

**Authors:** Junhao Huang, Jinglin Huang, Ziqing Liu, Xinyue Yang

**Affiliations:** 1 Guangdong Provincial Key Laboratory of Physical Activity and Health Promotion, Guangzhou Sport University, 1268 Middle Guangzhou Avenue, Guangzhou 510500, China; jinglin991012@163.com (J.H.); 15578838535@163.com (Z.L.); cynthiayoo33@gmail.com (X.Y.); 2 School of Exercise and Health, Shanghai University of Sport, Shanghai 200438, China

**Keywords:** exercise, executive function, fNIRS, sedentary behaviour

## Abstract

**Highlights:**

**What are the main findings?**

- Both HIIE and MICE reduced reaction times in the congruent and incongruent Stroop, 3-back, and single and mixing Switching trials; only HIIE reduced Stroop interference reaction time.
- Both modes increased circulating BDNF and lactate and produced task-specific prefrontal Oxy-Hb increases. HIIE elicited a larger lactate increase and additional increases in the Stroop FPA, Switching PTR, and N-back R-DLPFC.

**What are the implications of the main findings?**

- These findings motivate adequately powered head-to-head comparisons but do not establish that either exercise mode is superior across executive-function outcomes.
- Associations among lactate, BDNF, Oxy-Hb, and reaction time are exploratory and do not establish causal mechanisms.

**Abstract:**

Background/Objectives: To compare the acute effects of high-intensity interval exercise (HIIE) and moderate-intensity continuous exercise (MICE) on executive-function performance, prefrontal oxyhaemoglobin (Oxy-Hb) signals, brain-derived neurotrophic factor (BDNF), and lactate in sedentary young adults. Methods: Fifteen participants (10 men, 5 women; 22 ± 2 years; BMI 23.07 ± 4.12 kg/m^2^) completed HIIE, MICE, and seated control sessions in randomized order with 7-day washouts. Stroop, N-back, and Switching tasks; prefrontal fNIRS; plasma BDNF; and blood lactate were assessed before and after each condition. Correlation and indirect-effect analyses were exploratory. Results: Both HIIE and MICE significantly improved inhibitory control, working memory, and cognitive flexibility relative to baseline. For inhibitory control, both exercise modes reduced reaction times in congruent and incongruent Stroop tasks, but only HIIE improved Stroop interference performance. Both HIIE and MICE improved inhibitory control, working memory, and cognitive flexibility relative to baseline. Both reduced reaction times in the congruent and incongruent Stroop, 3-back, and Switching trials, whereas only HIIE improved Stroop interference. Both exercise modes increased Oxy-Hb in the OFA during Stroop and in the FPA and R-DLPFC during Switching. HIIE additionally increased Oxy-Hb in the FPA during Stroop, PTR during Switching, and R-DLPFC during N-back. Both exercise modes increased circulating BDNF and lactate, with a greater lactate response after HIIE. Improvements in executive function were associated with changes in Oxy-Hb, BDNF, and lactate. Conclusions: Both exercise sessions were followed by faster reaction times on selected tasks. The small sample, multiple analyses, and potential systemic contributions to fNIRS signals preclude firm conclusions about exercise-mode superiority or biological mediation.

## 1. Introduction

Sedentary behaviour (SB), defined by the World Health Organization as any waking behaviour performed while sitting, reclining, or lying with an energy expenditure ≤ 1.5 metabolic equivalents [1], is common in modern society [2,3]. Excessive SB and insufficient physical activity are associated with metabolic and cardiovascular disorders and poorer cognitive health [4,5]. Young adults may accumulate prolonged sitting during study and work [2]. Although cognition generally peaks in early adulthood, executive function (EF), including inhibitory control, working memory, and cognitive flexibility, may still vary with habitual activity [6]. Longer SB duration has been associated with poorer executive performance, although evidence in young adults remains limited [7].

Regular physical activity is recommended for health promotion [8], but the acute cognitive effects of different exercise modes remain uncertain. High-intensity interval exercise (HIIE) has been proposed as a time-efficient alternative to continuous aerobic exercise [9], whereas intensity-response accounts suggest that moderate-intensity continuous exercise (MICE) may also support cognition [10,11,12]. Direct comparisons are limited and show outcome- and population-specific patterns. Tsukamoto et al. reported larger postexercise executive-function effects after HIIE than after MICE in young adults [13], while Ahmadi et al. found similar immediate inhibitory-control responses but a larger delayed switching response after HIIE in older adults [14]. These findings support direct comparison without assuming that HIIE is uniformly superior.

Exercise-related changes in EF may involve task-related prefrontal haemodynamics, which can be examined with functional near-infrared spectroscopy (fNIRS) [15,16]. Acute exercise has been associated with altered dorsolateral prefrontal cortex (DLPFC) Oxy-Hb signals during executive tasks [17,18]. Lactate may also act as a metabolic and signalling molecule linked to BDNF-related pathways [19], and the previously reported parent study found a larger lactate response after HIIE than after MICE [20]. These measures can be temporally associated, but an acute crossover study cannot by itself establish a causal biological pathway.

Therefore, this study compared the acute effects of HIIE and MICE on EF performance, task-related prefrontal Oxy-Hb signals, BDNF, and lactate in sedentary young adults. We hypothesized that HIIE would produce larger changes than MICE, and we treated the correlation and indirect-effect analyses as exploratory tests of associations among the measured changes.

## 2. Materials and Methods

### 2.1. Participants and Study Design

The sample size was estimated a priori for the previously published parent study [20] using G*Power version 3.1 (F test, repeated-measures ANOVA). The calculation assumed an effect size f of 0.31, an alpha level of 0.05, statistical power (1 − beta) of 0.80, three experimental conditions, and two measurements (pre and post). The parent study reported a minimum requirement of 10 participants for its randomized crossover design. Fifteen participants (10 men and 5 women) completed the present study. Because the calculation was based on the vascular and endothelial outcomes of the parent study and was not designed for the cognitive, fNIRS, correlation, or indirect-effect analyses reported here, these outcomes were interpreted as exploratory rather than confirmatory. The study was approved by the Ethics Committee of Guangzhou Sport University (approval code: 2021LCLL-26; approval date: 20 December 2021), and all participants provided written informed consent. A single-blind, randomized crossover design was used. Each participant completed HIIE, MICE, and seated control (CON) in random order, with a 7-day washout between conditions. An independent experimenter labelled the conditions A, B, and C and generated the sequence using a random-number generator. Participants were informed of the assigned condition only after completing the baseline assessment for that session.

### 2.2. Experimental Procedure

**Figure 1:**
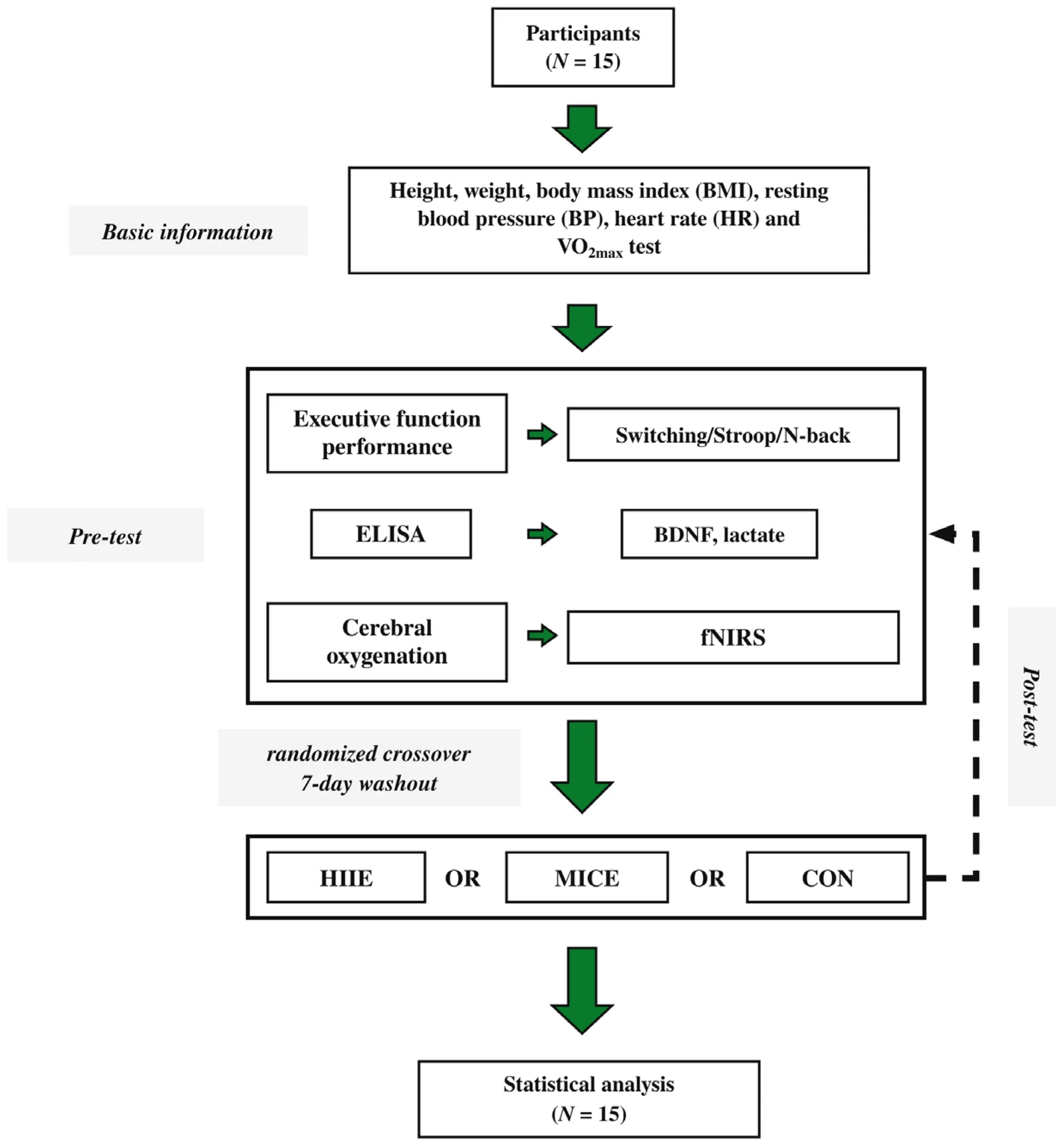
Experimental procedure.

One week before the formal intervention, all participants were instructed to wear comfortable exercise clothing and shoes to the laboratory for a maximal oxygen uptake (VO_2_max) test to determine individual optimal exercise intensities. To minimize the effects of biorhythms, all three main experiments were scheduled at the same time of day for each participant. Participants were instructed to fast for two hours before each formal intervention. After completing each pre-intervention baseline test, participants were informed of the upcoming intervention content. Each intervention was then completed under the supervision and guidance of a professional trainer. All tests and interventions were conducted at the Guangdong Provincial Key Laboratory of Physical Activity and Health Promotion. The experimental procedure is illustrated in Figure 1.

### 2.3. Measurement of VO_2_max

Participants completed a graded exercise test on a cycle ergometer (Ergoselect 100, ergolineGmbH, Bitz, Germany) at 60 rpm, starting at 55 W with 15 W increments every 2 min until exhaustion. VO2max was confirmed when at least two of the following criteria were met: oxygen uptake plateau, respiratory quotient ≥1.1, heart rate exceeding age-predicted maximum (220 − age), and Borg rating ≥19. The final-minute workload at each stage was used to establish individual standard curves, from which workloads corresponding to 20%, 40%, 65%, and 80% VO2max were calculated.

### 2.4. Exercise Protocols

The exercise protocols have been described previously [20]. Briefly, HIIE comprised a 2 min warm-up at 40% VO_2_max, 8 × 1 min intervals at 80% VO_2_max separated by 1 min active recovery at 40% VO_2_max, and a 3 min cool-down at 40% VO_2_max. MICE consisted of a 2 min warm-up at 20% VO_2_max, 30 min of continuous exercise at 60% VO_2_max, and a 3 min cool-down at 20% VO_2_max. Total work output was matched between protocols. During CON, participants remained seated for 1 h, avoided physical activity and sleep, and were allowed only sedentary activities such as reading or watching TV.

### 2.5. EF Performance Tasks

Reaction time was used as the principal behavioural measure because it can be sensitive to within-person cognitive change [21]. Accuracy was monitored and remained above 90%. The 3-back reaction time and the cognitive load effect were treated as distinct outcomes: the former represents performance at the highest task load, whereas the latter is the difference between 3-back and 1-back reaction time.

*Inhibitory control*: The Stroop task included congruent, neutral, and incongruent conditions. In the experiment, two rows of stimuli were presented on the screen. Participants indicated whether the colour of the top row of Chinese characters matched the meaning of the bottom row. The ‘F’ key was pressed for incongruent trials, and the ‘J’ key was congruent trials. In the congruent condition, the font colour matched the word meaning. In the incongruent condition, they mismatched. The Stroop interference effect was calculated by subtracting the congruent task reaction time from incongruent task reaction time.

*Working memory*: The N-back task used an increasing memory load paradigm (N = 1, 2, 3) to assess working memory capacity, with digits 0–9 as stimuli. Participants compared the current digit with the digit presented N trials earlier according to the memory load level. The cognitive load effect was calculated by subtracting 1-back reaction time from 3-back reaction time.

*Cognitive flexibility*: Switching tasks were used to assess cognitive flexibility, reflecting the ability to shift attention between tasks with different rules. The task comprised two parts: a single trial task and a mixing trial task. The single trial task included two subtasks: Task A (judge the size of a red number) and Task B (judge the parity of a green number). In the mixing trial task, stimuli appeared in a random sequence following an “ABBAABBA” pattern, requiring participants to switch tasks based on the colour of the numbers presented.

### 2.6. Measurements of Circulating Biomarkers

At the beginning and the end of each experiment, approximately 1–2 mL of fingertip blood was collected using K^+^-EDTA anticoagulant tubes and centrifuged at 3500 rpm for 10 min at 4 °C. Plasma samples were stored in a −80 °C for later analysis. Plasma BDNF concentration was determined using an ELISA kit (Cusabio, Wuhan, China) according to the manufacturer’s instructions. Optical density was measured at 450 nm wavelength using a microplate reader (Bio Tek Instruments, Winooski, VT, USA). Lactate levels were measured using a handheld lactate tester (Lactate Scout, SensLab, Leipzig, Germany).

### 2.7. Cerebral Oxygenation

A multichannel NIRSport portable near-infrared spectroscopy system (NIRx Medical Technologies, LLC, Glen Head, NY, USA) measured task-related changes in prefrontal oxygenated haemoglobin (Oxy-Hb). Participants were instructed to remain still, minimize facial expressions and head movements, and wear a visor to reduce external-light interference. The system used wavelengths of 760 and 850 nm, eight sources, seven detectors, 20 channels, and a sampling rate of 7.81 Hz. Channels were grouped into five predefined scalp-level regions of interest (ROIs): orbitofrontal area (OFA; CH01, CH02), frontopolar area (FPA; CH03, CH04, CH05, CH08, CH09), pars triangularis (PTR; CH13, CH15, CH16, CH17), left dorsolateral prefrontal cortex (L-DLPFC; CH06, CH07, CH12, CH18, CH19), and right dorsolateral prefrontal cortex (R-DLPFC; CH10, CH11, CH14, CH20). No short-separation channels were used, and heart rate, blood pressure, respiration or end-tidal carbon dioxide, skin or scalp blood flow, and scalp perfusion were not recorded concurrently. These ROI labels describe approximate cortical projections; the measured Oxy-Hb signal may therefore include systemic and extracerebral components.

### 2.8. Statistical Analysis

Data were analysed using SPSS 22.0 and are presented as mean ± standard deviation (SD). After assessment of the distributional assumptions, a two-way repeated-measures ANOVA (Time: pre, post × Condition: HIIE, MICE, CON) was fitted separately for each behavioural outcome, biomarker, and task-specific fNIRS ROI. Where applicable, pairwise post hoc contrasts within a fitted model were Bonferroni adjusted. Pearson correlations were restricted to Stroop interference, cognitive load effect, and mixing-trial reaction time and their corresponding biomarker and Oxy-Hb change scores. Indirect-effect models were estimated with PROCESS using 5000 bootstrap samples and bias-corrected 95% confidence intervals (CIs). An indirect-effect CI excluding zero was described as a statistical indirect association, not as evidence of causal mediation. No additional familywise-error or false-discovery-rate correction was applied across the full set of behavioural outcomes, ROI/task combinations, correlations, or indirect-effect models. Correlation and indirect-effect *p* values are therefore nominal and should be interpreted as exploratory.

For fNIRS analysis, MATLAB version 2018b was used for preprocessing and motion-artifact correction. Signals were band-pass filtered at 0.01–0.08 Hz, followed by wavelet minimum description length detrending and low-pass filtering based on the haemodynamic response function. At the individual level, a general linear model estimated task-related Oxy-Hb responses and generated beta values for each channel and condition. Beta values were averaged across channels within each ROI. Because no short-separation channels or concurrent systemic physiological recordings were available, short-separation regression and adjustment for systemic covariates were not performed. The beta values are therefore reported as Oxy-Hb signals rather than localized neural activation.

## 3. Results

### 3.1. EF Performance

During Stroop task (Table 1), reaction times in the congruent task decreased significantly after HIIE and MICE (*p* < 0.01 and *p* < 0.05, respectively) but not after CON compared to baseline. Meanwhile, reaction times in the incongruent task also decreased after both HIIE and MICE (both *p* < 0.001) but not after CON. Moreover, reaction times in the incongruent task were significantly lower in HIIE and MICE compare to CON (*p* < 0.001 and *p* < 0.01, respectively). However, Stroop interference reaction time decreased significantly only after HIIE condition (*p* < 0.05), not after MICE or CON.

During the N-back task (Table 1), 3-back reaction time decreased from baseline after HIIE and MICE (*p* < 0.001 and *p* < 0.05, respectively) but not after CON. In contrast, 1-back reaction time, 2-back reaction time, and the cognitive load effect did not change in any condition (all *p* > 0.05). Thus, the observed 3-back response should not be interpreted as a change in the derived cognitive load effect.

During Switching task (Table 1), reaction times in single trial task decreased significantly after HIIE and MICE (both *p* < 0.01) but not after CON. Similarly, reaction times in the mixing trial task decreased after HIIE and MICE (*p* < 0.001 and *p* < 0.01, respectively) but not after CON.

### 3.2. Cerebral Oxygenation

During Stroop task (Table 2), Oxy-Hb levels in the OFA region increased significantly after HIIE and MICE (*p* < 0.01 and *p* < 0.05, respectively) but not after CON. Oxy-Hb in the FPA region increased significantly only after HIIE (*p* < 0.05). No changes were observed in the PTR, L-DLPFC, or R-DLPFC regions in any condition (all *p* > 0.05).

During N-back task (Table 2), Oxy-Hb in the R-DLPFC region increased significantly only after HIIE (*p* < 0.05). No changes were observed in other regions across conditions (all *p* > 0.05).

During Switching task (Table 2), Oxy-Hb in the FPA region increased significantly after both HIIE and MICE (both *p* < 0.05) but not after CON. Oxy-Hb in the PTR and R-DLPFC regions increased significantly only after HIIE (both *p* < 0.05). No changes were observed in the OFA or L-DLPFC regions (all *p* > 0.05).

### 3.3. Circulating Biomarker Levels

**Figure 2:**
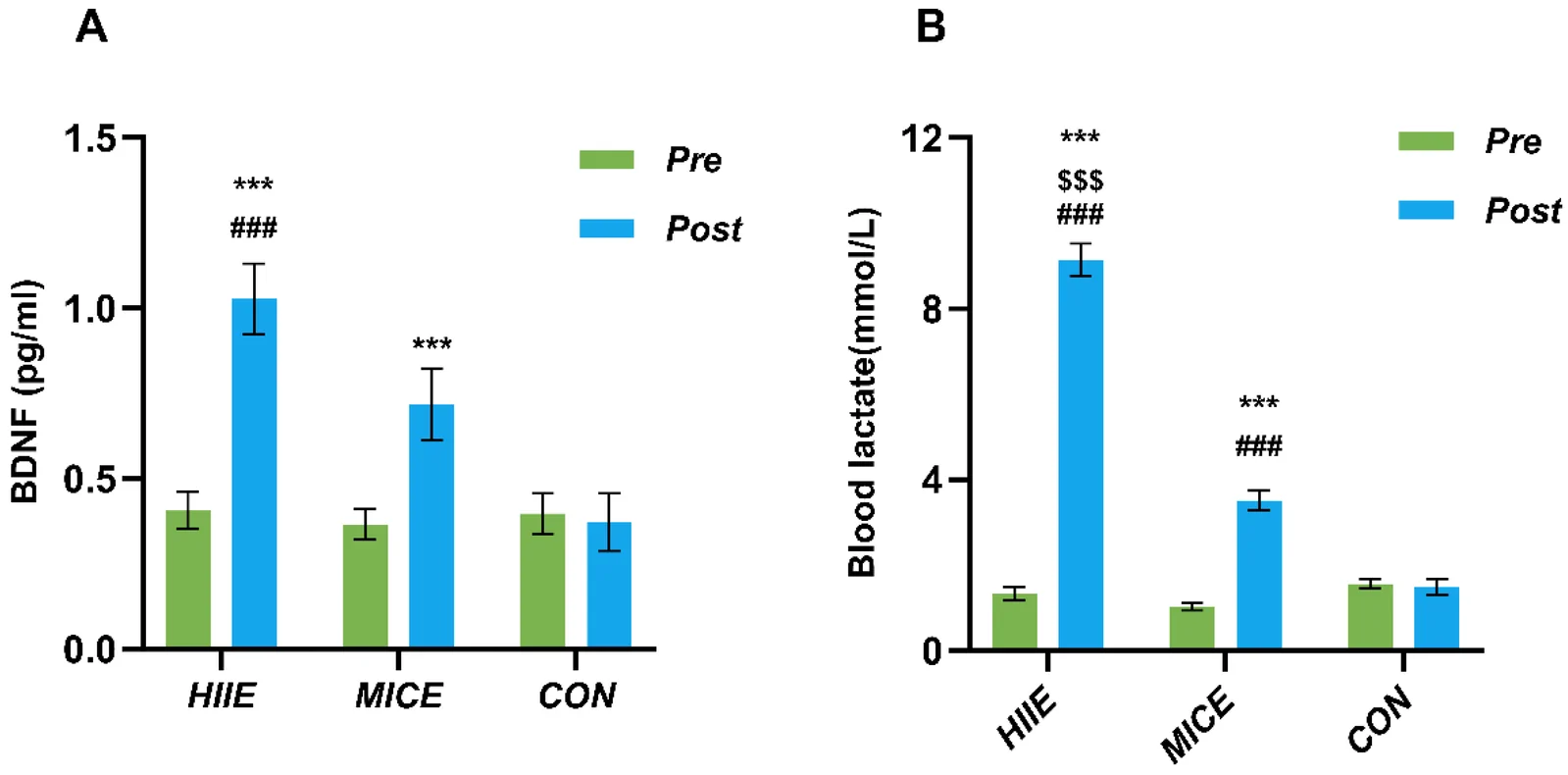
Effects of acute HIIE and MICE on circulating BDNF and lactate levels in sedentary young individuals. The statistical analyses showed the BDNF level (**A**) and the lactate level (**B**) in the pre- and post-visits of HIIE, MICE, and CON conditions. Values are the mean ± SEM. *** *p* < 0.001 vs. pre-visit; ^$$$^ *p* < 0.001 vs. MICE; ^###^ *p* < 0.001 vs. CON. HIIE: high-intensity interval exercise; MICE: moderate-intensity continuous exercise; CON: control.

As shown in Figure 2A, circulating BDNF levels increased significantly after HIIE and MICE (both *p* < 0.001) but not after CON. Moreover, BDNF levels were also significantly higher after HIIE compared to CON (*p* < 0.001). Lactate levels enhanced significantly after both HIIE and MICE (both *p* < 0.001) but not after CON (Figure 2B). Furthermore, lactate levels were significantly higher after both HIIE and MICE compared to CON (both *p* < 0.001) with a greater increase after HIIE than after MICE (*p* < 0.001).

### 3.4. Exploratory Correlation and Indirect-Effect Analyses

**Figure 3:**
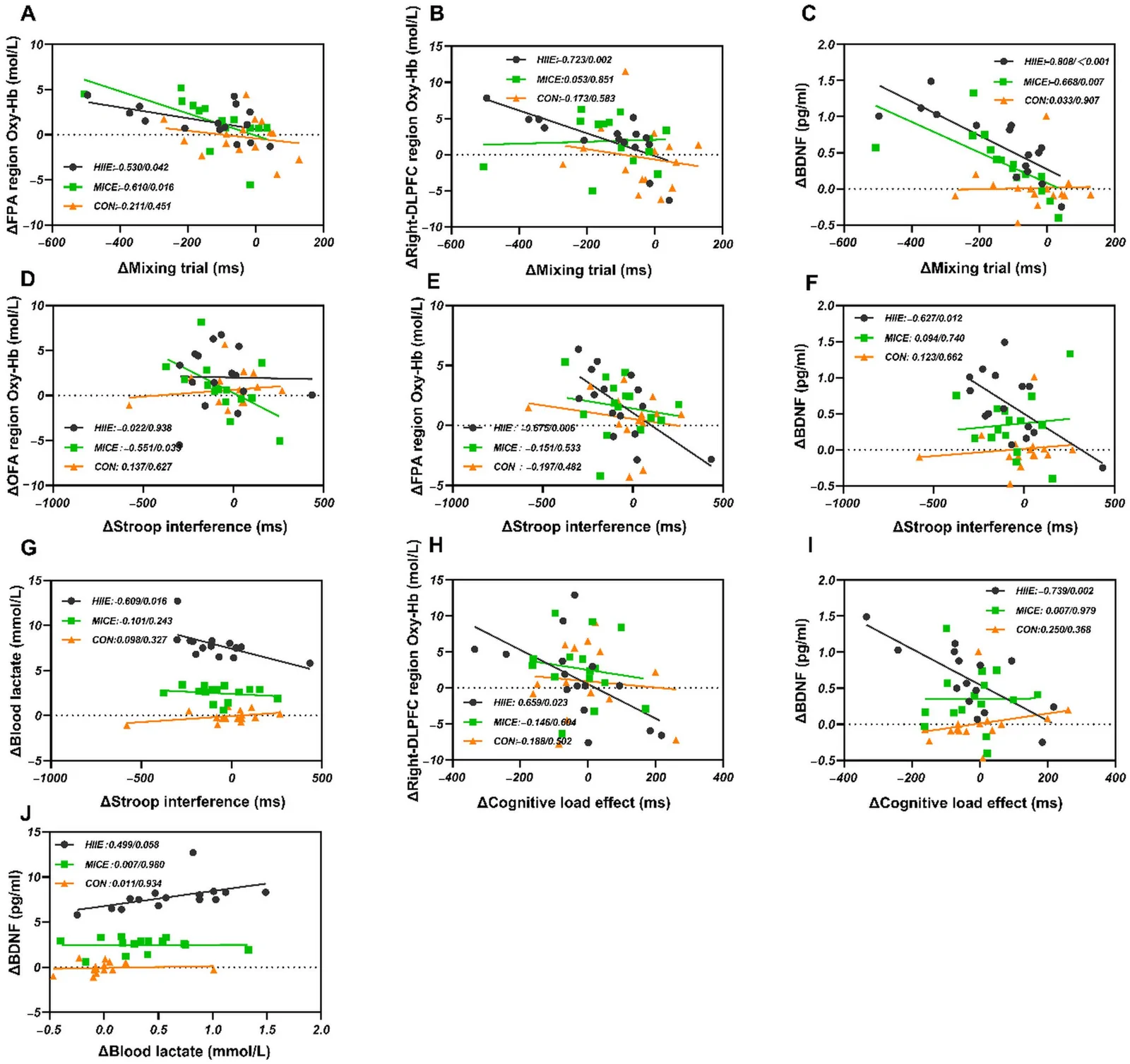
Results of Pearson’s correlation analysis. Pearson correlation analyses of change scores for mixing-trial reaction time with FPA Oxy-Hb (**A**), R-DLPFC Oxy-Hb (**B**), and BDNF (**C**); Stroop interference reaction time with OFA Oxy-Hb (**D**), FPA Oxy-Hb (**E**), BDNF (**F**), and blood lactate (**G**); cognitive load effect with R-DLPFC Oxy-Hb (**H**) and BDNF (**I**); and BDNF with blood lactate (**J**). Coefficients and *p* values are nominal and were not adjusted across the full correlation set. OFA: orbitofrontal area; FPA: frontopolar area; DLPFC: dorsolateral prefrontal cortex.

In the exploratory analyses, change in OFA Oxy-Hb was nominally inversely correlated with change in Stroop interference reaction time after MICE (Figure 3D). Changes in FPA Oxy-Hb, BDNF, and lactate were nominally inversely correlated with change in Stroop interference reaction time after HIIE (Figure 3E–G). These correlations were not adjusted across the full set of tests.

After HIIE, changes in R-DLPFC Oxy-Hb and BDNF were nominally inversely correlated with change in the cognitive load effect (Figure 3H,I). The cognitive load effect itself did not change significantly after HIIE, MICE, or CON. These associations therefore describe interindividual covariation in change scores and are not evidence that either intervention improved the cognitive load effect.

**Figure 4:**
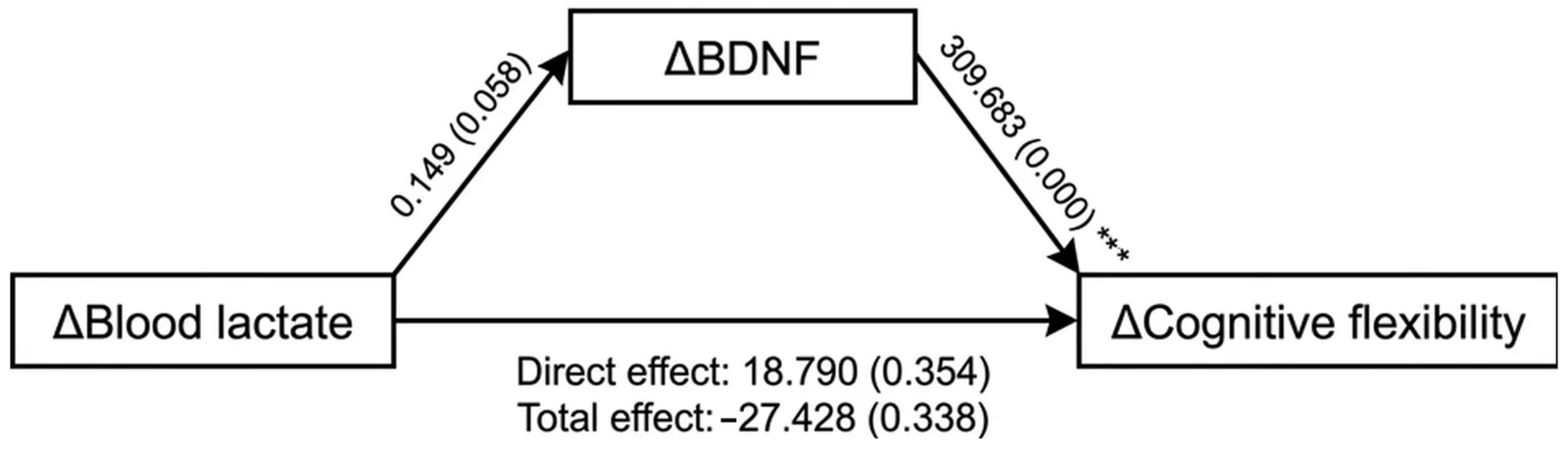
The exploratory indirect-effect analysis. The model estimated the indirect association between change in blood lactate and change in mixing-trial reaction time through change in BDNF in the HIIE condition. Values are unstandardized regression coefficients with *p* values. *** *p* < 0.001. The model is exploratory and does not establish temporal or causal mediation.

For the Switching task, changes in FPA Oxy-Hb and circulating BDNF were nominally inversely correlated with change in mixing-trial reaction time after both HIIE and MICE (Figure 3A,C). Change in R-DLPFC Oxy-Hb was nominally inversely correlated with change in mixing-trial reaction time after HIIE (Figure 3B), and changes in BDNF and lactate were correlated after HIIE (Figure 3J). In the HIIE condition, the bootstrapped CI for the indirect association from lactate change to mixing-trial reaction-time change through BDNF change excluded zero (Figure 4). Given the small sample, contemporaneous change scores, and absence of correction across models, this result does not establish causal mediation.

## 4. Discussion

This randomized crossover study compared energy-matched HIIE and MICE across behavioural, prefrontal Oxy-Hb, and circulating biomarker outcomes in 15 sedentary young adults. Both exercise sessions were followed by shorter reaction times in congruent and incongruent Stroop trials, the 3-back task, and single and mixing Switching trials. Only HIIE showed a within-condition reduction in Stroop interference reaction time, while the cognitive load effect did not change. Selected task-related Oxy-Hb signals increased after exercise, with more regional within-condition changes after HIIE. BDNF and lactate increased after both modes, and the lactate response was larger after HIIE. This pattern does not establish overall HIIE superiority because the study was small, tested many outcomes, and did not demonstrate a consistently larger between-condition effect across executive-function measures.

For inhibitory control, congruent and incongruent Stroop reaction times decreased after both exercise modes, whereas Stroop interference reaction time decreased only after HIIE. A statistically significant within-HIIE change combined with a nonsignificant within-MICE change is not, by itself, evidence that the two exercise effects differ. De Diego-Moreno et al. likewise reported task-specific responses when comparing high-intensity functional exercise with MICE [22]. Acute exercise-related cognitive changes have been associated with prefrontal haemodynamic responses [23], and the prefrontal cortex supports inhibitory control [24]. In the present study, changes in Oxy-Hb, BDNF, lactate, and Stroop interference reaction time were correlated in selected conditions. Because these were uncorrected change–score correlations, they are best viewed as hypotheses for future work rather than evidence of a lactate–BDNF–prefrontal mechanism.

Several physiological processes could contribute to the observed postexercise pattern. Acute exercise alters arousal, cardiac output, ventilation, vascular tone, substrate availability, and circulating neurotrophic factors. Vascular function can influence cerebral haemodynamics [25], and preclinical studies link exercise-related lactate exposure with BDNF signalling [19,26]. However, this study did not measure cerebral blood flow, catecholamines, or central BDNF release. Peripheral lactate and plasma BDNF therefore cannot identify the direction or location of a causal pathway.

For working memory, 3-back reaction time decreased after HIIE and MICE, which indicates faster responding at the highest tested load. The derived cognitive load effect (3-back minus 1-back reaction time) did not change in any condition and should not be described as improved. The R-DLPFC Oxy-Hb signal increased after HIIE but not after MICE or CON. Previous exercise studies and lesion work support a role for the DLPFC in working-memory performance [27,28,29]. In this sample, change in R-DLPFC Oxy-Hb and BDNF correlated with change in the cognitive load effect after HIIE. Because the cognitive load effect showed no intervention-related change and the correlations were uncorrected, these results do not demonstrate that R-DLPFC oxygenation or BDNF mediated a working-memory benefit.

For cognitive flexibility, single- and mixing-trial reaction times decreased after HIIE and MICE. FPA and R-DLPFC Oxy-Hb signals increased after both exercise modes, whereas PTR Oxy-Hb increased only after HIIE. The DLPFC and frontopolar cortex contribute to cognitive flexibility and complex task control [30,31]. Nominal correlations linked changes in mixing-trial reaction time with FPA Oxy-Hb and BDNF after both exercise modes and with R-DLPFC Oxy-Hb after HIIE. The HIIE indirect-effect model was statistically compatible with an indirect association through BDNF, but the model used contemporaneous change scores from 15 participants. It cannot determine temporal ordering or show that lactate caused BDNF release or cognitive change.

The Oxy-Hb findings also require physiological qualification. fNIRS signals can contain cerebral, scalp, and systemic components, and exercise can alter heart rate, blood pressure, respiration, carbon dioxide, skin blood flow, and scalp perfusion. These influences can produce apparent task-related Oxy-Hb changes that are not specific to local neural activity [32]. Because no short-separation channels or concurrent systemic physiological measures were collected, the regional results should be interpreted as prefrontal Oxy-Hb signals rather than localized neural activation.

The study was not powered to evaluate sex differences: only five participants were women, and no sex-by-condition analysis was performed. Sex-related variation is plausible because the acute BDNF response to exercise has differed with sample sex composition in meta-analysis [33], and cerebrovascular responsiveness can vary by sex and menstrual phase [34]. The pooled estimates should therefore not be interpreted as identical responses in men and women. Future studies should recruit balanced samples and prospectively account for menstrual phase and hormonal contraceptive use where relevant.

Several limitations constrain interpretation. First, the sample comprised 15 participants, including only five women. This limits precision, makes correlations and indirect effects unstable, and precludes reliable sex-stratified inference. Second, many behavioural outcomes, task-specific fNIRS ROIs, correlations, and indirect-effect models were examined. Bonferroni adjustment was limited to pairwise contrasts within fitted models; no study-wide familywise-error or false-discovery-rate correction was applied. Nominal associations may therefore include false-positive findings. Third, the cognitive load effect did not change despite a decrease in 3-back reaction time, so its correlations should not be used as evidence of an intervention-related working-memory improvement. Fourth, the fNIRS analysis could not separate cerebral activity from systemic or extracerebral effects. Fifth, the acute design cannot establish the temporal chain required for biological mediation or predict long-term adaptation. Finally, the findings may not generalize beyond sedentary young adults. Larger, preregistered studies with prespecified primary outcomes, systemic physiological monitoring, short-separation fNIRS channels, and balanced recruitment are needed.

## 5. Conclusions

In conclusion, acute HIIE and MICE were each followed by faster reaction times on selected executive-function tasks in sedentary young adults. HIIE showed a broader pattern of within-condition behavioural and prefrontal Oxy-Hb changes and produced a larger lactate response, but the present data do not establish its overall superiority. Associations among lactate, BDNF, Oxy-Hb signals, and reaction time are hypothesis-generating and do not demonstrate causal mediation. These findings warrant confirmation in larger studies with prespecified outcomes and improved control of systemic fNIRS influences.

## Figures and Tables

**Table 1:** Effects of acute HIIE and MICE on reaction time (ms) of cognitive tasks in sedentary young individuals.

Task		HIIE	MICE	CON	Time	Condition
Stroop						
Congruent	Pre	752.23 ± 128.96	754.33 ± 137.71	761.63 ± 121.18	0.008	0.147
	Post	638.05 ± 158.11 **	667.76 ± 116.54 *	768.98 ± 89.77		
Incongruent	Pre	854.82 ± 81.64	857.24 ± 91.70	858.17 ± 89.60	<0.001	0.008
	Post	664.54 ± 113.81 ***###	707.02 ± 120.54 ***##	844.71 ± 105.88		
Stroop interference	Pre	102.59 ± 160.89	102.91 ± 186.64	96.55 ± 113.22	0.047	0.864
	Post	26.48 ± 93.12 *	39.25 ± 153.25	75.73 ± 133.42		
N-back						
1-back	Pre	526.91 ± 152.45	510.31 ± 91.22	519.81 ± 98.67	0.11	0.314
	Post	451.31 ± 80.87	480.46 ± 83.20	556.64 ± 111.90		
2-back	Pre	604.53 ± 131.71	600.51 ± 148.83	606.61 ± 119.62	0.921	0.99
	Post	594.76 ± 126.43	609.20 ± 134.47	601.15 ± 114.82		
3-back	Pre	627.41 ± 124.97	621.53 ± 76.70	618.07 ± 96.67	0.003	0.165
	Post	523.15 ± 92.11 ***	573.22 ± 77.10 *	652.29 ± 95.40		
Cognitive load effect	Pre	100.51 ± 151.03	111.22 ± 104.27	98.26 ± 115.65	0.346	0.907
	Post	71.83 ± 83.85	92.76 ± 97.68	95.65 ± 131.32		
Switching						
Single trial	Pre	605.44 ± 176.81	597.27 ± 118.90	600.71 ± 142.96	0.001	0.305
	Post	501.84 ± 91.62 **	494.06 ± 81.84 **	617.05 ± 123.85		
Mixing trial	Pre	764.35 ± 205.52	747.30 ± 163.94	779.31 ± 155.31	<0.001	0.26
	Post	614.68 ± 130.68 ***	618.94 ± 116.26 **	737.37 ± 177.71		

Values are the mean ± SD. * *p* < 0.05, ** *p* < 0.01, *** *p* < 0.001 vs. pre-visit; ## *p* < 0.01, ### *p* < 0.001 vs. CON. HIIE: high-intensity interval exercise; MICE: moderate-intensity continuous exercise; CON: control.

**Table 2:** Effects of acute HIIE and MICE on task-related prefrontal Oxy-Hb signals (10^−4^ mol/L) in sedentary young adults.

Regions		HIIE	MICE	CON	Time	Condition
Stroop						
OFA	Pre	−0.18 ± 1.98	−0.20 ± 2.49	−0.23 ± 0.38	<0.001	0.559
	Post	1.65 ± 1.81 *	1.37 ± 1.97 *	0.38 ± 2.19		
FPA	Pre	−0.44 ± 2.41	−0.38 ± 2.86	−0.23 ± 1.27	0.009	0.519
	Post	1.58 ± 1.54 *	0.54 ± 1.37	0.36 ± 1.92		
PTR	Pre	−0.23 ± 2.43	−1.54 ± 3.16	0.14 ± 1.86	0.040	0.198
	Post	0.62 ± 1.76	0.19 ± 2.46	0.90 ± 3.27		
L-DLPFC	Pre	−0.66 ± 1.43	−0.26 ± 3.38	0.10 ± 2.62	0.449	0.080
	Post	−0.00 ± 2.43	−0.99 ± 1.97	1.41 ± 2.71		
R-DLPFC	Pre	−1.18 ± 2.86	0.70 ± 3.17	0.57 ± 0.93	0.648	0.441
	Post	0.43 ± 4.23	−0.44 ± 2.56	2.95 ± 2.90		
N-back						
OFA	Pre	0.55 ± 2.22	0.20 ± 2.56	0.27 ± 2.61	0.657	0.53
	Post	0.63 ± 2.57	0.06 ± 3.26	−0.43 ± 1.42		
FPA	Pre	0.14 ± 1.28	0.37 ± 1.68	0.08 ± 2.24	0.416	0.858
	Post	0.63 ± 1.92	0.57 ± 1.99	0.41 ± 1.38		
PTR	Pre	0.14 ± 1.69	0.70 ± 2.66	−0.36 ± 2.32	0.451	0.502
	Post	0.11 ± 1.37	0.65 ± 2.21	0.88 ± 2.50		
L-DLPFC	Pre	0.58 ± 3.18	1.07 ± 2.31	0.38 ± 2.18	0.439	0.253
	Post	−0.37 ± 1.29	1.04 ± 3.66	0.02 ± 2.06		
R-DLPFC	Pre	−0.48 ± 3.23	−0.40 ± 3.62	−0.50 ± 3.56	0.040	0.548
	Post	2.18 ± 3.13 *	0.83 ± 3.30	0.46 ± 3.52		
Switching						
OFA	Pre	−0.28 ± 2.15	−0.08 ± 3.35	−0.05 ± 1.88	0.366	0.775
	Post	1.01 ± 1.96	0.13 ± 2.49	−0.06 ± 2.82		
FPA	Pre	−0.17 ± 1.54	−0.43 ± 1.94	−0.20 ± 1.56	0.008	0.134
	Post	1.36 ± 1.70 **	1.05 ± 1.78 *	−0.43 ± 1.46		
PTR	Pre	−0.46 ± 2.11	−0.24 ± 2.42	−0.21 ± 2.20	0.040	0.849
	Post	1.12 ± 1.58 *	0.21 ± 2.15	0.41 ± 2.64		
L-DLPFC	Pre	0.54 ± 1.71	0.94 ± 2.90	0.38 ± 2.61	0.642	0.435
	Post	0.91 ± 1.93	0.53 ± 3.45	−0.38 ± 2.65		
R-DLPFC	Pre	0.31 ± 2.06	0.40 ± 1.98	0.49 ± 2.65	0.036	0.081
	Post	2.47 ± 2.32 *	2.29 ± 2.52 *	0.11 ± 2.73		

Values are mean ± SD. The displayed *p* values are the main effects of time and condition from the repeated-measures ANOVA. * *p* < 0.05, ** *p* < 0.01 versus pre-session. HIIE: high-intensity interval exercise; MICE: moderate-intensity continuous exercise; CON: control; OFA: orbitofrontal area; FPA: frontopolar area; PTR: pars triangularis; L-DLPFC: left dorsolateral prefrontal cortex; R-DLPFC: right dorsolateral prefrontal cortex.

## Data Availability

The data presented in this study are available on request from the corresponding author due to participant privacy considerations.

## Abbreviations

The following abbreviations are used in this manuscript:

BDNF	Brain-derived neurotrophic factor
BMI	Body mass index
CH	Channel
DLPFC	Dorsolateral prefrontal cortex
EF	Executive function
fNIRS	Functional near-infrared spectroscopy
FPA	Frontopolar area
HIIE	High-intensity interval exercise
L-DLPFC	Left dorsolateral prefrontal cortex
MICE	Moderate-intensity continuous exercise
OFA	Orbitofrontal area
Oxy-Hb	Oxygenated haemoglobin
R-DLPFC	Right dorsolateral prefrontal cortex
SB	Sedentary behaviour
